# Recombination of Porcine Reproductive and Respiratory Syndrome Virus: Features, Possible Mechanisms, and Future Directions

**DOI:** 10.3390/v16060929

**Published:** 2024-06-07

**Authors:** Xing-Yang Cui, Da-Song Xia, Ling-Zhi Luo, Tong-Qing An

**Affiliations:** 1 State Key Laboratory for Animal Disease Control and Prevention, Harbin Veterinary Research Institute, Chinese Academy of Agricultural Sciences, Harbin 150069, China; 2 College of Animal Science, Wenzhou Vocational College of Science and Technology, Wenzhou 325006, China

**Keywords:** PRRSV, recombination, viral evolution, vaccine safety

## Abstract

Recombination is a pervasive phenomenon in RNA viruses and an important strategy for accelerating the evolution of RNA virus populations. Recombination in the porcine reproductive and respiratory syndrome virus (PRRSV) was first reported in 1999, and many case reports have been published in recent years. In this review, all the existing reports on PRRSV recombination events were collected, and the genotypes, parental strains, and locations of the recombination breakpoints have been summarized and analyzed. The results showed that the recombination pattern constantly changes; whether inter- or intra-lineage recombination, the recombination hotspots vary in different recombination patterns. The virulence of recombinant PRRSVs was higher than that of the parental strains, and the emergence of virulence reversion was caused by recombination after using MLV vaccines. This could be attributed to the enhanced adaptability of recombinant PRRSV for entry and replication, facilitating their rapid propagation. The aim of this paper was to identify common features of recombinant PRRSV strains, reduce the recombination risk, and provide a foundation for future research into the mechanism of PRRSV recombination.

## 1. Introduction 

Porcine reproductive and respiratory syndrome (PRRS), characterized by reproductive failure in sows and respiratory dysfunction in pigs of all ages, has caused tremendous economic losses to the global swine industry [1,2]. PRRS emerged in North America in 1987 [3] and Europe in 1990 [4]. The causative agent, PRRS virus (PRRSV), is a single-stranded positive-sense RNA virus belonging to the genus Betaarterivirus, family Arteriviridae, order Nidovirales. PRRSV is classified into two species: Betaarterivirus suid 1 (formerly PRRSV-1 or European-type PRRSV) and Betaarterivirus suid 2 (formerly PRRSV-2 or North American-type PRRSV) [5]. The genomes of these two PRRSV species share about a 60% nucleotide similarity [6]. The PRRSV genome contains at least 11 open reading frames (ORFs), including ORF1a, ORF1b, ORF2a, ORF2b, ORF5a, and ORFs 3–7 [7]. ORF1a and ORF1b encode replicase polyproteins that are post-translationally cleaved into at least 16 non-structural proteins (NSP) [8,9], whereas ORF2a, ORF2b, ORF5a, and ORFs 3-7 encode the viral structural proteins GP2, E, ORF5a, GP3, GP4, GP5, M, and N, respectively [10]. 

The PRRSV genome exhibits extensive genetic variations owing to high-frequency mutations, insertions/deletions, and recombination. Based on the phylogenetic analysis of the ORF5 gene and the global PRRSV classification system, PRRSV-1 is divided into at least three subtypes: subtype 1, subtype 2, and subtype 3 [11]. Initially, PRRSV-1 was only detected in European swine herds. At present, PRRSV-1 has been observed in North American and Asian swine herds [12,13,14,15,16]. In 2011, PRRSV-1 was reported in mainland China from clinical samples [12]. Since then, there have been occasional reports of PRRSV-1 on pig farms in China. PRRSV-2 is the dominant PRRSV genotype in North America and Asian countries. The significant genetic variation in PRRSV-2 has also led to the classification of PRRSV-2 strains into 11 lineages, lineages 1 to 11 (L1–L11), based on the ORF5 gene [17,18]. Over the past 30 years, a number of new PRRSV variants have emerged in most pig-raising countries, including the highly pathogenic PRRSV (HP-PRRSV) variant, which emerged in China in 2006 and was characterized by a unique discontinuous deletion of 30 amino acids (aas) in NSP2 [19]. From there, the HP-PRRSV spread rapidly to more than ten Asian countries [20]. Additionally, an NADC30 strain was first reported in the US in 2008, characterized by a unique discontinuous 131 aa deletion in NSP2 [21]. Subsequently, the NADC30-like PRRSV was imported into China, where it underwent recombination with the native HP-PRRSV [22,23]. In 2020, recombinant PRRSV RFLP 1-4-4 L1C emerged in the US. Researchers at the University of Minnesota detected recombinants using a time-scaled phylogenetic analysis, proving the complex recombination scheme of the highly pathogenic L1C-1-4-4 strain [24]. The L1C-1-4-4 variant caused many miscarriages and mummified fetuses in pregnant sows, along with the death of adult sows. In addition, researchers at Iowa State University demonstrated the mismanagement of two commercial vaccines (Ingelvac and Prevacent) through the systematic use of next-generation sequencing (NGS) and whole genome sequencing (WGS). A novel pathogenic PRRSV recombinant strain was observed in the USA, and weaned piglets infected with recombinant strains exhibited symptoms such as high fever, with the mortality rate reaching 15–30% [25]. In recent years, more than a dozen recombinant PRRSV variants with different pathogenicity have been reported [26,27,28,29]. Moreover, some modified live vaccines (MLVs) against PRRS have undergone recombination with wild-type PRRSVs [29,30,31,32], causing serious concern in the swine industry. 

Recombination in RNA viruses is characterized as the exchange of genetic material between at least two distinct viral genomes. When two or more viruses co-infect the same cell, genomic fragments may be exchanged, leading to recombination. Recombination is a major driver of genetic shifts associated with the rapid evolution of RNA viruses, facilitating the emergence of new viral variants by altering their cellular tropism, antigen profiles, and pathogenicity. Additionally, recombination in RNA viruses can have several effects, such as the emergence of virulent variants, the generation of deficient viruses, the evasion of host RNAi defense, and the potential influence on vaccine efficacy [33]. Most RNA viruses recombine through homologous or nonhomologous recombination, both of which play key roles in the biology and evolution of RNA viruses. Recombination is a pervasive phenomenon in RNA viruses and an important strategy for accelerating the evolution of RNA viral populations. Furthermore, recombination has been widely observed in positive-strand RNA viruses, including severe acute respiratory syndrome-coronavirus-2 (SARS-CoV-2) [34,35], poliovirus [36], Western equine encephalitis virus [37], hepatitis C virus [38], foot-and-mouth disease virus [39], and PRRSV [40]. The research on RNA recombination helps predict the probability and outcome of viral recombination events and design viruses with lower recombination frequencies as candidate viruses for developing attenuated live vaccines. In addition, the surveillance of viral recombination should be prioritized when detecting viral strain outbreaks to better determine the disease epidemic trend and its prevention.

Recombination hotspots were identified as regions exhibiting the highest recombination rates and frequencies within the genome. Recombination hotspots within sequences can be used to elucidate evolutionary processes and the consequences of recombination. The recombination position was determined solely based on the approximate position obtained from the analysis of the representative reference strain. The breakpoints were randomly distributed because the recombination process is random. However, the survival and replication of recombinant viruses may be subject to natural selection pressures. Only a portion of the recombinant viruses can survive, with the population of recombinant viruses exhibiting enhanced entrance or replication abilities that expand during viral proliferation.

The recombination of PRRSV has been reported for several years, with most of them being limited to case reports. The understanding of PRRSV recombination is fragmented, and there is a lack of a systematic view of PRRSV recombination.

## 2. Recombination of PRRSV

### 2.1. Clinical Events of Recombinant PRRSV

PRRSV recombination was initially documented in 1999 in Marc-145 cells, a PRRSV-permissive African green monkey kidney cell line [41]. Then, van Vugt et al. showed that PRRSV recombination can occur between diverse European isolates under cell culture conditions, but no recombination was detected between the European and North American types [42]. For PRRSV-1, Forsberg et al. first reported the recombination observation of wild-type PRRSV-1 in Italy in 2001 [43]. Subsequently, Fang et al. found a novel isolate, 04-41, during the genetic diversity and evolution analysis of PRRSV-1 isolates collected in North America. A phylogenetic tree and SimPlot analysis showed that the isolate 04-41 was a product of recombination [13]. The above seminal reports on PRRSV-1 recombination laid an important foundation for the subsequent research. PRRSV-1 recombination between two field strains was mainly reported in some European countries, such as Italy [43], Britain [44], and the Netherlands [45]. The recombination of PRRSV-1 has been also reported in North American and Asian countries, such as the United States [13] and China [46,47]. Moreover, recombination between the PRRSV-1 field strains and the PRRSV-1 MLV strains has been reported in some European countries, including France [26] and Hungary [48]. Similar events have been reported in Asian countries, such as China [49]. Recently, instances of recombination between two PRRSV-1 MLV strains have been reported; for example, the PRRS-FR-2014-56-11-1 strain has emerged as a recombinant between the Unistrain vaccine (VP-046BIS strain; Hipra) and the Porcilis vaccine (DV strain; MSD) [50]. Additionally, the PRRSV Horsens strain has emerged as a result of recombination between the Amervac MLV and the 96V198 MLV strains, causing an epidemic in Danish pig farms [27]. 

According to the existing published reports on PRRSV recombination, the number of PRRSV-2 recombination reports is higher than that of PRRSV-1. Based on these data, we inferred that the PRRSV-2 recombination events were more frequent than those for PRRSV-1, largely owing to the higher prevalence of PRRSV-2 in the pig population. According to the global recombinant analysis of PRRSV-2, recombinant PRRSV-2 has been mainly detected in China and the US, with smaller numbers detected in Korea [51]. Interestingly, the recombinant hotspot appears to be associated with the pig-raising density, particularly in regions with a high pig density within the state/province, that is, Henan, Guangdong, and Shandong in China or Iowa and Minnesota in the US. This phenomenon can be primarily attributed to the prevalence of multi-lineages of the PRRSV epidemic in the high-density pig-raising area, facilitating viral co-infection within pig farms and creating opportunities for viral recombination.

### 2.2. Distribution of Major or Minor Parent PRRSV Lineage

**Figure 1:**
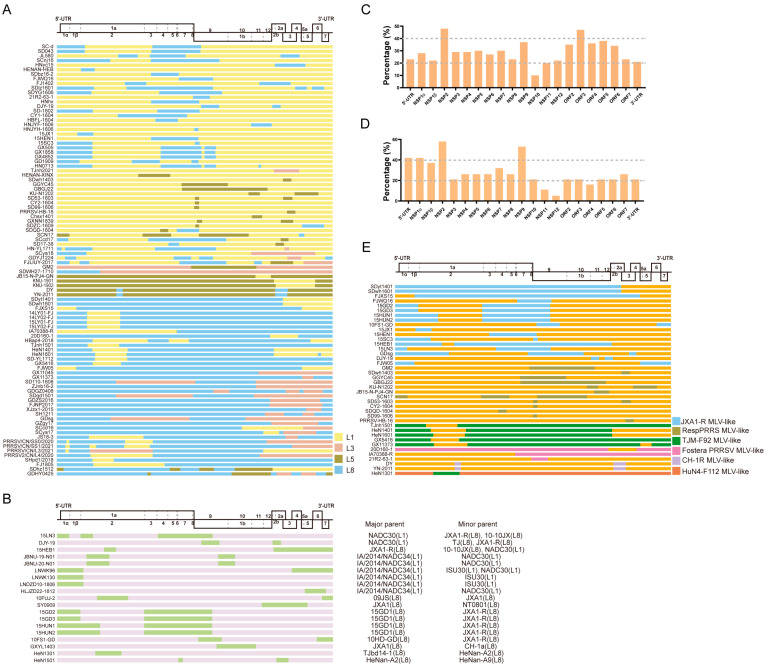
The recombination patterns of all the reported PRRSV-2 recombinant cases. The diagram of the full-length genome structure includes the major ORFs’ positions and boundaries, referring to VR-2332. (**A**) The inter-lineage and (**B**) intra-lineage recombination model diagram. Each junction of different colors means the breakpoints and indicates that the recombination occurs in this position. The different colors on the right indicate the parental lineages. The yellow rectangle represents the major parent, while the green rectangle box shows the minor parent. The recombination frequency of each coding or non-coding region in the (**C**) inter-lineage and (**D**) intra-lineage recombinant PRRSVs. The x-axis shows the position of the PRRSV-2 genome, and the y-axis represents the percentage of recombination events that occurred in the specific composition of the recombinant genome. (**E**) The MLV-related recombination patterns of PRRSV-2. The different colors of the box depict the different PRRS MLV vaccines shown on the right, and orange indicates all the other parents who are unrelated to the MLV vaccine. The major and minor parents are shown on the right, and the identified lineages are labeled.

PRRSVs exhibit significant genetic variation, resulting in the classification of PRRSV-1 and PRRSV-2 into several lineages based on their ORF5 genes [17]. At present, due to the small number of subtypes of PRRSV-1, it is currently divided into three or four subtypes [11,40,52,53]; therefore, the number of inter-lineage recombination events may be less. Moreover, the number of existing reports on PRRSV-1 recombination is also small. Analyses with less data may not be able to obtain some regular feature information about recombination; therefore, in this part, we only discuss the recombination event of PRRSV-2. In recent years, frequent intra-/inter-lineage recombination of PRRSV-2 has occurred as summarized in Figure 1 (The detailed color of representative PRRS strain for recombination is shown in Table S1). Inter-lineage recombination has been widely reported (Figure 1A), and the prevalence of L1, L3, and L8 PRRSVs exhibited extensive recombination. For instance, NADC30-like PRRSV (SC-d) is a recombinant between the JXA1-like and NADC30-like strains and is classified as an inter-lineage recombination between L1 and L8 [54]. The strains SH1211 and GD1404 were recombinants between L3 and L8 [55,56]. Moreover, SD043 is a recombinant strain isolated from China that twice underwent recombination events, with its potential parental strains being L1 and L8 [57]. Combined with the current reports on PRRSV recombination and the data analysis results from our laboratory [51,58], we observed a gradual change in the recombination pattern of PRRSV inter-lineage recombination. L1 PRRSV was found to be susceptible to recombination among PRRSVs both in China and the US. The major recombination pattern shifted from an L8 to an L1 backbone between 2014 and 2018 for Chinese PRRSVs, whereas L1 consistently remained the major backbone for US PRRSVs [58]. Genetic analyses have revealed that the population genetic diversity of NADC30-like PRRSV is declining annually, whereas the genetic diversity of NADC34-like PRRSV is increasing [59,60]. Given that the NADC30-like and NADC34-like strains are members of the L1 PRRSV family, we speculate that L1 PRRSV will continue to dominate the recombination pattern between PRRSV recombinants. The major parents were the L1 and L8 strains for the inter-lineage recombinant strains. However, with the continuous evolution of PRRSV in recent years, the major parents of recombinant PRRSV strains are mainly L1. 

For intra-lineage recombination (Figure 1B), the recombination of PRRSV occurs mainly in L1 and L8, whereas in the US, intra-lineage recombination was observed only within the L1 PRRSV. Recently, a novel RFLP 1-4-4 L1C variant, which is a recombinant virus obtained by exchanging the ORF1a gene fragment between L1C and L1A, emerged in the US [24]. Similarly, two South Korean strains, JBNU-19-N01 and JBNU-20-N01, are intra-lineage recombinants of the L1 PRRSVs [61]. The NADC34-like strains LNDZD10-1806 and HLJZD22-1812, isolated in China, were also derived from recombination within L1 PRRSVs [62]. 

The recombination frequency of each coding or non-coding region in the PRRSV was statistically analyzed. For inter-lineage recombination, the results highlight that the ORF3, NSP9, and NSP2 segments were replaced most frequently (Figure 1C). Moreover, for intra-lineage recombination, the majority of the recombinant strains isolated in recent years are associated with recombination events within the L1 PRRSV, particularly involving the genome locations of fragment exchange in NSP9 and NSP2 (Figure 1D). In the earlier years, intra-recombination was mainly dominated by L8 PRRSV.

### 2.3. Distribution of Recombinant Breakpoints

The recombinant breakpoint distribution was investigated to explore the recombination characteristics of the PRRSV further. All the case reports on PRRSV-2 recombination were collected, and the recombination breakpoints were identified and analyzed (Figure 1A,B). Combined with the previous statistical and analytical results of PRRSV recombination events in our laboratory [46,51,58], these results demonstrate that PRRSV-2 does have hotspots for recombination, whereas the hotspot is variable in different recombination patterns. When L1 PRRSV was the major parental strain involved in recombination, the hotspots were mainly located in the NSP regions, such as NSP2 and NSP9. In contrast, when the major parental recombinant strains were L8 PRRSVs, the recombination hotspots were mainly dispersed within the structural protein regions, such as ORF2-4 and ORF5. The above conclusions are based on the rules summarized by the collected recombination data. Moreover, it should be noted that the sample collection was random and may not fully reflect the actual recombination differences. Interestingly, most recombinants with L8 PRRSVs as the backbone retained the NSP9 region of HP-PRRSV (Figure 1A). In our previous study, we comprehensively analyzed all the available PRRSV-2 genomic sequences (n = 941) from 1991 to 2021 and found that the majority of the recombinant breakpoints were concentrated in the NSP9 and ORF2-4 regions [51,58]. 

In order to further analyze the recombination features of PRRSV-1, 88 complete genome sequences (isolated in 19 countries) from 1991 to 2018 in GenBank and three PRRSV-1 strains (HL85, HeB3, and HeB47) isolated from clinical samples in 2018 were collected. A recombination analysis of the 91 available genomic sequences was performed by SimPlot and RDP in our laboratory, and finally, 24 recombinant strains were obtained, and all the recombinants belonged to subtype 1. Similar results were obtained for the recombination hotspot of PRRSV-1; the high-frequency recombination regions of the identified PRRSV-1 recombinants were distributed in NSP2 and ORF2-4, for which the recombination frequency was greater than or equal to 25%, and five recombination events involving vaccine strains [46]. Other recombinant strains of PRRSV-1 have similar characteristics, such as the PRRSV-1 isolate 04-41, and the recombination analysis identified a breakpoint located downstream of the NSP2/3 junction [13]. The breakpoint of ‘Horsens virus’ DK-2019-10166-107 is also located in ORF3 [27].

Notably, NSP2 is the most variable protein; however, it did not affect the pathogenicity of PRRSV [63]. GP2 and GP4 interact with the CD163 receptor and are the major determinants of PRRSV entry into cells [64]. NSP9 encodes an RNA replicase of PRRSV, which is closely related to PRRSV replication [65]. The recombination of PRRSV occurring in ORF2-4 or NSP9 may be associated with increased cellular tropism or replication efficiency, potentially facilitating easier infection or replication of the recombinant PRRSV. 

### 2.4. PRRS MLV Vaccine-Related Recombination 

Currently, several commercial PRRS MLV vaccines have been reported to be associated with recombination events, including Ingelvac PRRS MLV, Fostera PRRS MLV, and some HP-PRRS MLVs (Figure 1E). For instance, the SCN17 strain is a recombinant of RespPRRS MLV, NADC30-like PRRSV, and JXA1-like PRRSV. Piglets infected with SCN17 can develop persistent fever, interstitial pneumonia, and viremia [29]. The recombinant TJnh1501 strain is the product of recombination between JXA1-P80 MLV and NADC30-like PRRSV. Pathogenicity tests showed that piglets inoculated with TJnh1501 exhibit fever, respiratory symptoms, and significant pathological lung damage [30]. The GDsg strain recombined with the low-pathogenic wild-type strains QYYZ and JXA1-P80, resulting in a higher pathogenicity than the QYYZ strain [31]. In 2018, a recombinant PRRSV IA70388-R between the Fostera PRRS MLV and a field strain was isolated in Iowa, providing solid evidence for recombination between an MLV strain and a circulating strain in the US [32]. The Fostera PRRS MLV was detected in South Korea in 2014 and has also undergone recombination with field PRRSV in South Korea [66]. Recombination of the PRRSV-1 wild-type strain and vaccine strain has also been found in pig herds in Germany [67]. In recent years, recombination events have occurred between two PRRS MLV vaccine strains, such as the TJM-F92 MLV and HuN4-F112 MLV in China [68]. In addition to the PRRSV-2 vaccine strain, similar reports have been reported for the PRRSV-1 vaccine strain, such as the Unistrain MLV and Porcilis MLV in France [26] and the Amervac strain (Unistrain PRRS vaccine; Hipra) and 96V198 strain (SuvaxynPRRS; Zoetis AH) in Denmark [27]. Additionally, the recombination patterns related to PRRS MLV vaccines were analyzed in this study (Figure 1E). Interestingly, the recombination breakpoint was found to be mainly located in NSP2 and NSP9, which are associated with the modulation of the host inflammatory response or cellular replication of PRRSV [69,70,71]. JXA1-R was found to be primarily involved in the recombination events, followed by the RespPRRSV MLV and TJM-F92 MLV. Following the outbreak of HP-PRRSV in China, MLV vaccines against HP-PRRS have been generated after serial passages in the Marc-145 cells, such as JXA1-R, HuN4-F112, and TJM-F92 [72,73,74,75]. Compared to inactivated PRRS vaccines, MLV vaccines exhibit better efficiency and are widely employed. However, the emergence of virulence reversion caused by recombination after the use of MLV vaccines has become a challenge that must be addressed.

## 3. Consequences of PRRSV Recombination

### 3.1. Changing Viral Pathogenicity in Pigs

The recombination of PRRSV may contribute to the emergence of novel genotypes, the failure of vaccine protection, and even changes in pathogenicity [33]. From 2009 to 2010, the number of HP-PRRS cases increased significantly in China. A phylogenetic analysis demonstrated that recombination played a crucial role in the second outbreak of HP-PRRS [76]. Importantly, new recombinant PRRSVs have replaced other circulating strains, emerging as the dominant lineage in China [77]. In 2013, a highly pathogenic PRRSV JL580 strain was isolated in a farm where serious pig deaths occurred; the virus was a recombination product of NADC30-like PRRSV and HP-PRRSV [28]. Similarly, SC-d is a recombinant strain of NADC30 and JXA1 characterized by higher pathogenicity than the non-recombinant NADC30-like strain [54]. In 2019, an NADC30-like strain named SD043 that underwent recombination twice was isolated; the SD043-infected piglets showed high levels of viral shedding in the respiratory or digestive tracts [57]. 

Recombinant PRRSV may escape immunity from existing vaccines, posing a serious threat to PRRS vaccines. Although most recombinant strains of PRRSV cause increased virulence, the recombinant strain of PRRSV-1 isolated in Russia showed decreased virulence [78].

### 3.2. MLV Vaccine Use Associated with Virulence Reversion

Given the constant emergence of new or recombinant PRRSV variants, it is essential to prevent the introduction and spread of the virus. Vaccination against PRRS can help reduce the impact of the virus on the swine population. However, owing to the widespread use of MLV vaccines, significant safety issues arise, such as recombination between MLV and field strains, potentially leading to virulence reversion [79]. The Ingelvac PRRS MLV recombined with the QYYZ-like strain and generated a new variant, GM2. Animal experiments showed that the virulence of the GM2 strain was significantly higher than that of either the QYYZ strain or Ingelvac PRRS MLV, attributed to the significantly higher replication capability of the GM2 strain than that of its parent strains in vivo [80]. The FJWQ16 strain showed 20% mortality in pigs and was found to be a recombinant between PRRSV NADC30 and HP-PRRS MLV [81]. Similarly, FJXS15 and TJnh1501 are the result of recombination between the HP-PRRS MLV vaccine and NADC30-like strains, both of which preserve NSP9 and NSP10 of the HP-PRRS MLV [30,82]. The recombinant Horsens strain seems to be highly transmissible and has caused severe disease in infected herds; in subsequent monitoring, it was also found that the prevalence of this recombinant strain resulted in fewer liveborns and more stillborns in herds. The impact of this recombinant PRRSV-1 on productivity exceeded that typically seen in Danish herds infected with PRRSV-1 [27,83].

## 4. Possible Recombination Mechanism of PRRSV

**Figure 2:**
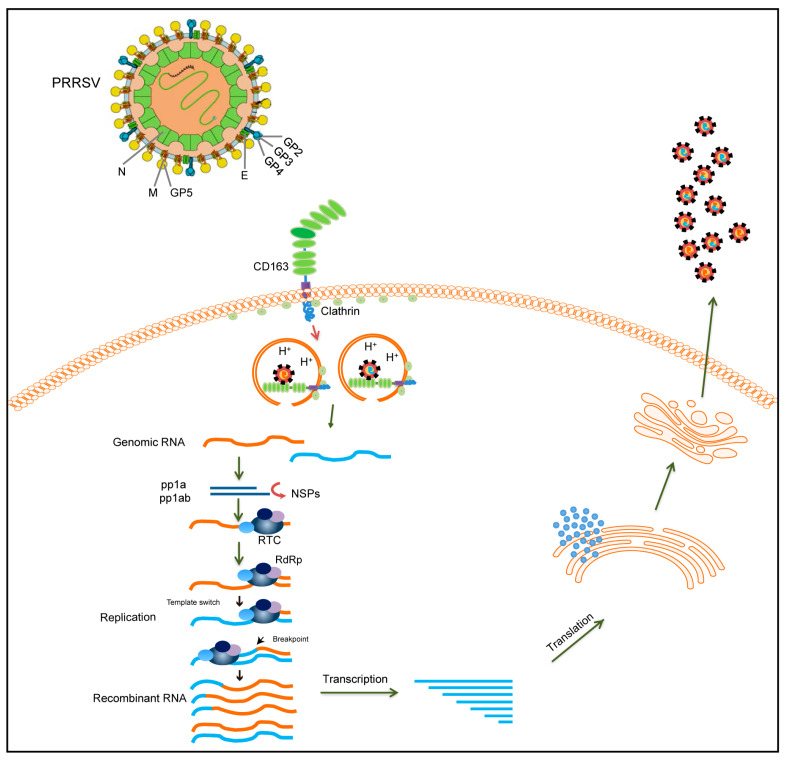
Schematic diagram illustrating the recombinant mechanism of PRRSV. During PRRSV infection, PRRSV enters the cell via the CD163 receptor. Different PRRSVs co-infect the same cell and can switch viral templates in replication. RdRp can switch from donor to receptor template to generate recombinant PRRSVs. The recombinant virion is finally released after transcription, translation, and assembly. The two different PRRSV genomes are represented by yellow and blue lines, respectively.

RNA viral recombination can be classified into three types: homologous recombination, aberrant homologous recombination, and nonhomologous recombination [84]. The replicative copy-choice model is a widely accepted mechanism of RNA–virus recombination. Most RNA viruses recombine via a copy-choice mechanism, during which RdRp switches from a donor to an acceptor template, resulting in the formation of recombinant RNA molecules [85]. The recombination mechanism of PRRSV may also conform to the copy-choice model (Figure 2). During viral replication, nidoviruses employ a discontinuous transcription mechanism to produce a nested set of 3′ co-terminal sgRNAs [86]. PRRSV is a member of the order Nidovirales, which has a similar transcription mechanism. According to the prevailing model of RNA synthesis in PRRSV, the body transcription-regulating sequence (TRS) in the 3′ region of the genome RNA is located immediately adjacent to the ORFs, and the body TRS (TRS-B) is identified by the leader TRS (TRS-L) during the generation of sgRNA. A TRS has also been demonstrated to play a role in the recombination of coronaviruses [87,88]. The RdRp pauses as it crosses the TRS-B and switches its template to the TRS-L. Furthermore, the secondary structure shows an exposed leader core sequence in the loop of a structured hairpin, which may enhance accessibility for hybridization with the TRS-B or contribute to recombination [89]. However, it remains unclear whether this mechanism also applies to PRRSV. To date, no feasible method has been developed to probe the roles of the TRS-B and TRS-L in PRRSV recombination. However, with the advancement of high-throughput sequencing technology, the detection of PRRSV recombination may become easier, and integrating sequencing technology and bioinformatic analysis could be a crucial tool for analyzing the relationship between PRRSV recombination and a TRS in the future. 

## 5. Factors Associated with the High Recombinant Ratio of PRRSV 

The recombination rate varied significantly among the various viral species. Positive-sense single-stranded RNA viruses such as arteriviruses, coronaviruses, and picornaviruses usually show a high ratio of recombination, whereas negative-sense single-stranded RNA viruses exhibit a low ratio of recombination [90]. The high recombination frequencies of PRRSV are associated with its widespread prevalence among pig populations and its ability to cause persistent viral infections [41]. Persistent infection may last for 4–5 months, during which the pigs may be reinfected with another PRRSV strain. Two or more strains infecting the same cell may have the opportunity for recombination. In addition, selective pressure may promote the recombination of PRRSV, as it has been reported that recombination can combine advantageous genes while removing deleterious mutations [91]. In contrast, PRRSV lacks the 3′ to 5′ exonuclease proofreading ability, resulting in a high mutation rate. While most mutations are harmful, the recombination events in PRRSV may slow the rate of accumulation of deleterious mutations. Additionally, the high ratio of intra-lineage recombination may be related to the high homology of the viral genome, as recombination in PRRSV occurs preferentially at positions with highly similar sequences [42]. Furthermore, a discontinuous transcription mechanism may promote PRRSV recombination. PRRSV sgRNAs are generated through discontinuous transcription, which is guided by the TRS-L and TRS-B. Template switching occurs when RdRp transcribes the negative strand and encounters the TRS-B [92]. The TRS usually correlates with the location of recombination breakpoints in coronaviruses [88], suggesting a similar situation may exist in PRRSV. 

The unreasonable use of PRRS MLV vaccines may also be one of the potential reasons for promoting PRRSV recombination. Since the emergence of PRRSV, more than ten MLV vaccines targeting PRRSV-1 or PRRSV-2 have been licensed in different countries and are widely used to prevent PRRS [79]. Notably, there is a period of viral shedding after inoculation with PRRS MLV vaccines during which field strains could recombine with MLV strains or recombination can occur between MLV strains [27,66]. Therefore, improper immunization programs may be responsible for the acceleration of PRRSV recombination. 

## 6. Summary and Future Directions

### 6.1. Summary

Recombination plays a crucial role in the expansion of the viral host range, emergence of new viral variants, changes in the range of transmission vectors, increases in virulence and pathogenicity, shifts in tissue propensity, evasion of the host immune response, and evolution of resistance to antiviral drugs [93]. RNA viruses frequently exploit recombination as a strategy for evolutionary adaptation, and increasing reports on PRRSV recombination across different regions or countries underscore the important role of recombination in the evolution of PRRSV [41,94,95,96]. Various MLV vaccines against PRRS are widely used in the pig industry. The occurrence of recombination events in MLV vaccines is a significant concern. Thus, the safety of PRRS MLV vaccines should be carefully assessed, and a cautious approach should be adopted for their practical application. Moreover, the potential of live-resident virus inoculation (LVI) to produce pathogenic recombinant strains is worth noting. LVI is an extremely risky practice common in the US and other parts of the world. 

Here, we systematically reviewed all the reported recombination events of PRRSV-1 and PRRSV-2 and determined their recombination characteristics, such as temporal, geographical, and parental recombination, and recombination breakpoint locations. We observed that the recombination pattern constantly changed regardless of whether it was inter- or intra-lineage recombination. The hotspots for recombination vary with the different patterns of recombination. The change in recombination patterns may be because only viruses adapted to the environment can survive under certain selection pressures and genetic evolution. Based on this finding, we may be able to infer the prevalence of the strain in the future and adopt appropriate control strategies for PRRS. Based on the analysis of the collected recombination events, we observed that the virulence of some recombinant PRRSVs was higher than that of the parental strains, which could be attributed to the enhanced adaptability of the recombinant PRRSV to entry and replication, facilitating their rapid propagation. In addition, virulence reversion is caused by recombination after the use of MLV vaccines. This phenomenon can provide a reference for the selection of vaccines for immunization prevention.

PRRSV takes the characteristics of persistent infection, the circulation of multi-lineage strains, and the widespread use of MLV vaccines to create opportunities for co-infection and contribute to viral recombination. We hypothesized that PRRSV may preferentially use the copy-choice replicative mode for recombination; however, further experiments are required to confirm this. These findings can be used to evaluate the prevalence and trends of PRRSV, which are conducive to preventing and controlling the disease on pig farms.

### 6.2. Future Directions on PRRSV Recombination

Although PRRSV recombination has been reported over the past few decades, current research focuses only on the recombination phenomenon and the characteristics of PRRSV; however, much remains to be studied. Some studies have reported that SARS-CoV-2 may have originated from recombination events [97]. SARS-CoV-2 has evolved and undergone recombination during its transmission. Both SARS-CoV-2 and PRRSV belong to the order Nidovirales and are prone to recombination, possibly related to their discontinuous transcriptional characteristics. The discontinuous transcription pattern of PRRSV and the generation of a nested set of 3′ co-terminal sgRNAs may pose challenges in determining recombination using sequencing technologies. Therefore, a suitable bioinformatics analysis to determine the recombination of PRRSV is required. In addition, few studies have directly addressed the mechanism of PRRSV recombination. The recombination mechanism of PRRSV is likely to comply with the copy-choice mode, but no research has been conducted to confirm it. Therefore, future studies must investigate these recombination mechanisms; methods are required to determine the factors influencing PRRSV recombination. High-throughput sequencing technology can facilitate comparative genomic studies based on mass genome sequences. Although these new technologies provide opportunities, significant challenges remain for several PRRSV recombination analyses. Therefore, developing effective high-throughput sequencing technology to analyze the PRRSV recombination process and characteristics is an important research direction.

So, safe PRRS MLV vaccines should be designed and developed. Certain recombination-related factors should be considered to reduce the risk of recombination. This implies that the modification of these motifs could aid in designing and developing recombination-deficient, genetically stable PRRS MLV strains. Several measures are recommended in pig production to reduce the risk of PRRSV recombination. First, from the perspective of vaccination, the use of different PRRS MLV vaccines should be avoided on the same farm, and MLV vaccines should be used rationally. Secondly, we attempted to reduce the risk of introducing new PRRSV strains into pig farms and the degree of viral contamination. Simultaneously, using MLV vaccines on breeding farms should be strictly prohibited to avoid the risk of viral exposure in closed herds. Third, reasonable production management practices should be implemented in pig farms, such as maintaining an appropriate density of pigs, employing a multipoint feeding model, and so on. 

PRRSV recombination is an extremely fascinating topic, and PRRSV could serve as a viral model to uncover the recombination mechanism of Nidovirales. Future studies on PRRSV recombination will contribute to a deeper understanding of the potential recombinant mechanism and viral evolution and may aid in the design and development of safer vaccines to mitigate the emergence of new recombinants. Related research on PRRSV recombination will add a global dimension to the significance of managing PRRSV infections in large swine populations. Moreover, it will benefit the prevention and control of pig diseases.

## Supplementary Materials

The following supporting information can be downloaded at: https://www.mdpi.com/article/10.3390/v16060929/s1, Table S1: The color of representative PRRS strain for recombination for Figure 1.
