# Supplementary material for: Recombination of Porcine Reproductive and Respiratory Syndrome Virus: Features, Possible Mechanisms, and Future Directions
Source: Viruses. 2024 Jun 7;16(6):929. doi: 10.3390/v16060929 (PMC11209122; doi:10.3390/v16060929)
Supplement: Supplementary file 1 [file viruses-16-00929-s001.zip › viruses-2995567-supplementary.pdf]

Table S1. The color of representative PRRS strain for recombination for Figure 1.

	Representative strain	Color	Diagram
Inter-lineage recombination	L1 PRRSV	Yellow	
	L3 PRRSV	Light pink	
	L5 PRRSV	Olive	
	L8 PRRSV	Blue	
Intra-lineage recombination	Major parent	Light pink	
	Minor parent	Light green	
MLV vaccine related recombination	JXA1-R MLV-like	Blue	
	RespPRRS MLV-like	Olive	
	TJM-F92 MLV-like	Green	
	Fostera PRRSV MLV-like	Deep pink	
	CH-1R MLV-like	Purple	
	HuN4-F112 MLV-like	Orange	
